# Draft Genome Sequence of *Marinobacter* sp. Strain AL4B, a Marine Bacterium Isolated from Quintero Bay, Chile

**DOI:** 10.1128/MRA.00856-21

**Published:** 2021-10-21

**Authors:** M. A. Dinamarca, K. González-Pizarro, M. Charifeh, P. Jopia, C. Ibacache-Quiroga

**Affiliations:** a Centro de Micro-Bioinnovación (CMBi), Universidad de Valparaíso, Valparaíso, Chile; b Escuela de Nutrición y Dietética, Facultad de Farmacia, Universidad de Valparaíso, Valparaíso, Chile; University of Southern California

## Abstract

Quintero Bay, located along the central coast of Chile, has suffered different oil spills during the past 10 years, impacting its marine ecosystems. Here, we report the genome sequence of *Marinobacter* sp. strain AL4B, a marine bacterium isolated from Quintero Bay, Chile.

## ANNOUNCEMENT

T he genus *Marinobacter* (1) belongs to the family *Alteromonadaceae*. *Marinobacter* species have been commonly isolated from diverse extreme marine and terrestrial environments (1–9). The genomic and phenotypic versatility of *Marinobacter* species enables them to colonize a wide variety of ecosystems, from psychrophilic to thermophilic marine environments, with high salinity and pH (6, 10, 11). Diverse species of *Marinobacter* are considered hydrocarbon-degrading microorganisms (1, 12, 13). Additionally, strains of *Marinobacter* species have been shown to produce biosurfactant compounds (14, 15) and present lipolytic activity (16–18).

Here, we report the genome sequence of *Marinobacter* sp. strain AL4B, belonging to the microbial community of an oil spill-impacted area in Quintero Bay in the Valparaíso Region, Chile (19).

*Marinobacter* sp. strain AL4B was isolated from seawater samples collected at a depth of 10 m. A water sample was taken from Quintero Bay (32°45′42″S, 71°29′44″W) by a diver using a sterile nuclease-free sampling bottle. Enrichment cultures were prepared by inoculating the sample in 250-ml Erlenmeyer flasks containing 100 ml of M9 minimal medium (12.8 g/liter Na_2_HPO_4_ × 7H_2_O, 3 g/liter KH_2_PO_4_, 0.5 g/liter NaCl, 10 g/liter NH_4_Cl, 10 mM MgSO_4_, 1 mM CaCl_2_), supplemented with 1% vol/vol of n-hexadecane (Sigma-Aldrich) as the sole energy and carbon source. The flasks were incubated at 25°C for 21 days. Pure cultures were obtained by streaking the enrichment cultures onto marine agar 2216 (Difco).

Genomic sequencing of *Marinobacter* sp. strain AL4B was carried out by Genoma Mayor SpA. For this purpose, strain AL4B was grown in marine broth 2216 (Difco) at 28°C and 200 rpm for 24 h. Bacterial DNA was extracted and purified using the DNeasy blood and tissue kit (Qiagen) according to the manufacturer’s instructions. DNA integrity was confirmed by agarose electrophoresis, and its concentration was determined by UV absorbance using the QuantiFluor fluorometer (Promega). DNA libraries were generated using the NEBNext Ultra II DNA library prep kit (New England Biolabs), and the genome was sequenced on the Illumina NovaSeq platform, using the paired-end protocol (2 × 150 bp). The sequence quality was analyzed using BBDuk software (part of the BBTools v. 38.91 package; https://sourceforge.net/projects/bbmap/). Illumina adaptors, low-quality bases on the right and left ends, and reads with Phred scores of <30 were trimmed. High-quality sequences were assembled using Megahit v. 1.2.8 software (20). The statistical analysis of the assembly was performed using QUAST v. 5.0.2 software (21), and a completeness analysis was carried out using BUSCO v. 5.2.2 software (22). The genome assembly was annotated using the NCBI Prokaryotic Genome Annotation Pipeline (PGAP) (23).

A total of 6,384,514 reads were generated from the sequencing process of *Marinobacter* sp. strain AL4B, with a coverage of 263×. The genome of strain AL4B has a total length of 3,639,286 bp, with a G+C content of 53.51% and 10 contigs (>500 bp). The contig *N*_50_ size is 1,286,268 bp. According to the analysis using NCBI PGAP, the genome of *Marinobacter* sp. strain AL4B contains 3,308 genes, 3,250 coding DNA sequences (CDSs), and 58 RNAs (10 rRNAs, 44 tRNAs, and 4 noncoding RNAs [ncRNAs]).

### Data availability.

The whole-genome sequence of *Marinobacter* sp. strain AL4B was submitted to the National Center for Biotechnology Information (NCBI) under GenBank accession number JAILWZ000000000.1 (assembly) and SRR15651962 (raw sequences).
